# Neuropeptides PDF and DH31 hierarchically regulate free-running rhythmicity in *Drosophila* circadian locomotor activity

**DOI:** 10.1038/s41598-018-37107-3

**Published:** 2019-01-29

**Authors:** Tadahiro Goda, Yujiro Umezaki, Fay Alwattari, Hanna W. Seo, Fumika N. Hamada

**Affiliations:** 1 Visual Systems Group, Abrahamson Pediatric Eye Institute, Division of Pediatric Ophthalmology, Cincinnati Children’s Hospital Medical Center, 3333 Burnet Avenue, Cincinnati, OH 45229 USA; 2 Division of Developmental Biology, Cincinnati Children’s Hospital Medical Center, Cincinnati, OH 45229 USA; 3 Department of Ophthalmology, College of Medicine, University of Cincinnati, Cincinnati, OH 45229 USA

## Abstract

Neuropeptides play pivotal roles in modulating circadian rhythms. Pigment-dispersing factor (PDF) is critical to the circadian rhythms in *Drosophila* locomotor activity. Here, we demonstrate that diuretic hormone 31 (DH31) complements PDF function in regulating free-running rhythmicity using male flies. We determined that *Dh31 loss-of-function* mutants (*Dh31*^*#51*^) showed normal rhythmicity, whereas *Dh31*^*#51*^*;Pdf*^*01*^ double mutants exhibited a severe arrhythmic phenotype compared to *Pdf-null* mutants (*Pdf*^*01*^). The expression of tethered-PDF or tethered-DH31 in clock cells, posterior dorsal neurons 1 (DN1ps), overcomes the severe arrhythmicity of *Dh31*^*#51*^*;Pdf*^*01*^ double mutants, suggesting that DH31 and PDF may act on DN1ps to regulate free-running rhythmicity in a hierarchical manner. Unexpectedly, the molecular oscillations in *Dh31*^*#51*^*;Pdf*^*01*^ mutants were similar to those in *Pdf*^*01*^ mutants in DN1ps, indicating that DH31 does not contribute to molecular oscillations. Furthermore, a reduction in *Dh31 receptor (Dh31r)* expression resulted in normal locomotor activity and did not enhance the arrhythmic phenotype caused by the *Pdf receptor (Pdfr)* mutation, suggesting that PDFR, but not DH31R, in DN1ps mainly regulates free-running rhythmicity. Taken together, we identify a novel role of DH31, in which DH31 and PDF hierarchically regulate free-running rhythmicity through DN1ps.

## Introduction

In *Drosophila*, pigment-dispersing factor (PDF) is required for robust locomotor behavioral output^1^. PDF coordinates circadian networks and controls the timing of morning and evening peaks in locomotor activity^2–6^. In the absence of PDF (*Pdf*^*01*^), flies show a loss of morning anticipation, an advanced evening activity peak, shorter free-running periods, and dampened molecular oscillations in clock cells^7–11^, suggesting that PDF is a main neuropeptide responsible for orchestrating the activity of each pacemaker neuron. However, the lack of PDF does not completely dampen free-running rhythm; approximately half of *Pdf*^*01*^ mutants still maintain weak rhythmic locomotor activity under constant dark (DD) conditions^9,10^. The data suggest that PDF may not be the only molecule responsible for regulating free-running rhythmicity. Therefore, we sought to identify another neuropeptide that complements the role of PDF in free-running rhythmicity.

In addition to PDF, diuretic hormone 31 (DH31) activates the PDF receptor (PDFR), which regulates locomotor activity^12^. DH31 is expressed in clock neurons in the brain. An RNA-seq analysis using sorted clock cells in the brain suggested that DH31 is expressed in lateral neurons (LNvs) and dorsal neurons 1 (DN1s)^13^, and DH31 antibody staining shows that DH31 is expressed in posterior dorsal neurons 1 (DN1ps)^14,15^. However, we and others have shown that *Dh31* mutants exhibit normal locomotor activity rhythms^14,15^.

Along with locomotor activity rhythms, DH31 plays roles in sleep and temperature preference rhythm (TPR). A recent study showed that PDF signaling is relayed to DN1s, which express DH31, to promote awakening at dawn^14^. We also recently showed that DH31 acts on dorsal neurons 2 (DN2s) via PDFR to modulate TPR, particularly the decrease in preferred temperature at the transition from day to night^15^. Therefore, we hypothesized that normal locomotor activity rhythms in *Dh31* single mutants might be a result of normal PDF signaling. To this end, we examined locomotor activity rhythms using *Dh31*-*Pdf* double mutants.

Here, we identify a novel role for DH31 in regulating the circadian rhythms of locomotor activity. We determined that PDF and DH31 hierarchically function to regulate free-running rhythmicity by acting on the same clock cells (DN1ps). These neuropeptides appear to play important roles in modulating the clock networks involved in free-running rhythmicity. Therefore, the identification of this novel DH31 function deepens our mechanistic understanding of the circadian rhythms of locomotor activity.

## Results

### DH31 is involved in regulating free-running rhythmicity

To re-evaluate the function of DH31 in regulating the circadian rhythms of locomotor activity, we focused on a double mutant of *Dh31*^*#51*^ (a *loss-of-function* mutant)^16^ and *Pdf*^*01*^ (a *null* mutant)^7^ and examined the phenotypes for rhythmicity, free-running period, morning anticipation and evening activity peaks.

**Figure 1:**
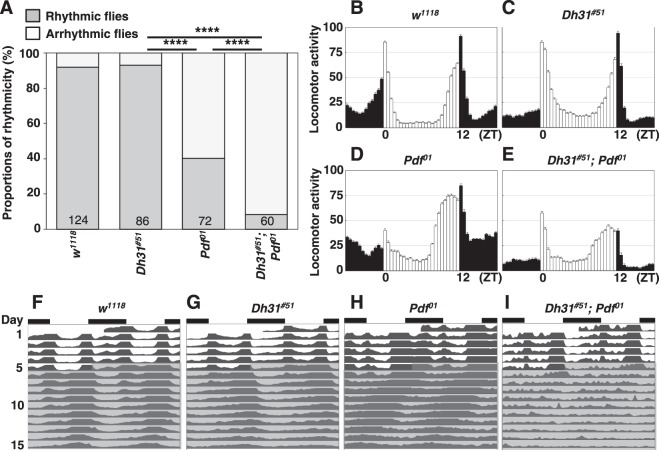
*Dh31*-*Pdf* double mutants exhibited severely disrupted free-running rhythmicity. (**A**) Comparison of free-running rhythms for different genotypes. The proportions of rhythmic (gray bar) and arrhythmic (white bar) flies over 10 days in DD were compared via χ^2^ analysis. ****P < 0.0001. Numbers in the bar graphs represent the number of flies. (**B**–**E**) Average daily actogram over 4 days in LD for each genotype: *w*^*1118*^ (**B**), *Dh31*^*#51*^ (**C**), *Pdf*^*01*^ (**D**), and *Dh31*^*#51*^*;Pdf*^*01*^ (**E**). (**F**–**I**) Double-plotted averaged actogram of rhythmic flies over 5 days in LD and 10 days in DD for each genotype: *w*^*1118*^ (**F**), *Dh31*^*#51*^ (**G**), *Pdf*^*01*^ (**H**), and *Dh31*^*#51*^*;Pdf*^*01*^ (**I**).

We determined that both *w*^*1118*^ (WT) and *Dh31*^*#51*^ mutant flies maintained a robust free-running rhythmicity (WT: 92% rhythmic, power = 1371.7, *Dh31*^*#51*^: 93% rhythmic, power = 678.2) (Fig. 1A,F,G and Table 1). These data are consistent with previous reports using *Dh31*^*#51*^ mutants^15^ and another *Dh31* mutant^14^. In contrast, the *Pdf*^*01*^ mutants exhibited a weak free-running rhythmicity (40% rhythmic, power = 243.0) (Fig. 1A,H and Table 1), which is also consistent with previous reports^9,10^. However, we determined that the free-running rhythmicity of *Dh31*^*#51*^*;Pdf*^*01*^ double mutants was strongly disrupted: 92% of the flies exhibited an arrhythmic phenotype, and only 8% showed weak amplitudes (power = 226.7) (Fig. 1A,I and Table 1). These data indicate that the *Dh31*^*#51*^*;Pdf*^*01*^ double-mutant phenotype is more severely arrhythmic than either single mutant, suggesting that DH31 is involved in modulating free-running rhythmicity.

**Table 1:** Free-running rhythms.

Genotype (DD1–10)	Total	Rhythmic	Tau (hr)		Power		Fig. #
	n	n (%)	Ave	SEM	Ave	SEM	
*w* ^*1118*^	124	114 (92)	24	0.02	1371.7	75.1	1,2,3,5,S4
*Dh31* ^*#51*^	86	80 (93)	24.4	0.06	678.2	46	1,2,3,S4
*Pdf* ^*01*^	72	29 (40)	22.5	0.12	243	32.5	1,2,3,S4
*Dh31*^*#51*^; *Pdf*^*01*^	60	5 (8)	23.1	0.9	226.7	37.3	1,2,3,S4
*tim-Gal4*/+, *Dh31*^*#51*^; *Pdf*^*01*^	102	20 (20)	23.2	0.12	406.6	42.3	2,3
*Pdf-Gal4*/+, *Dh31*^*#51*^; *Pdf*^*01*^	58	25 (43)	22.3	0.09	371.6	35.7	2,S4
*R18H11-Gal4*/+, *Dh31*^*#51*^; *Pdf*^*01*^	48	9 (19)	22.5	0.13	336.8	50.3	3,S4
+*/UAS-Dh31*, *Dh31*^*#51*^; *Pdf*^*01*^	110	21 (19)	22.6	0.11	409.8	49.5	2,S4
+*/UAS-Pdf*, *Dh31*^*#51*^; *Pdf*^*01*^	42	9 (21)	22.4	0.34	255.8	20.8	2
+*/UAS-t-Dh31*, *Dh31*^*#51*^; *Pdf*^*01*^	46	8 (17)	23.6	0.27	247.9	40.9	3,S4
+*/UAS-t-Pdf*, *Dh31*^*#51*^; *Pdf*^*01*^	47	4 (9)	22	0.44	277.2	43.8	3,S4
*tim-Gal4* > *UAS-Dh31*, *Dh31*^*#51*^; *Pdf*^*01*^	61	31 (51)	22.6	0.06	817.9	63.2	2
*tim-Gal4* > *UAS-t-Dh31*, *Dh31*^*#51*^; *Pdf*^*01*^	60	22 (37)	23	0.15	252.1	19.2	3
*tim-Gal4* > *UAS-t-Pdf*, *Dh31*^*#51*^; *Pdf*^*01*^	48	34 (71)	22.9	0.17	542.6	48.3	3
*Pdf-Gal4* > *UAS-Pdf*, *Dh31*^*#51*^; *Pdf*^*01*^	31	29 (94)	23.6	0.17	1043.6	109.5	2
*Pdf-Gal4* > *UAS-t-Dh31*, *Dh31*^*#51*^; *Pdf*^*01*^	59	25 (42)	22.9	0.15	307.5	34.8	S4
*Pdf-Gal4* > *UAS-t-Pdf*, *Dh31*^*#51*^; *Pdf*^*01*^	54	22 (41)	22.5	0.16	302.7	32.4	S4
*R18H11-Gal4* > *UAS-Dh31*, *Dh31*^*#51*^; *Pdf*^*01*^	73	20 (27)	22.5	0.11	487.4	63.7	S4
*R18H11-Gal4* > *UAS-t-Dh31*, *Dh31*^*#51*^; *Pdf*^*01*^	45	17 (38)	23.1	0.14	492.7	79.9	3
*R18H11-Gal4* > *UAS-t-Pdf*, *Dh31*^*#51*^; *Pdf*^*01*^	41	34 (83)	22.2	0.16	506.1	46.3	3
*Pdfr* ^*5304*^	55	28 (51)	23.3	0.08	323.6	29.4	5
*Dh31r* ^*1/Df*^	61	57 (93)	24	0.05	1033	81.22	5
*Pdfr*^*5304*^; *Dh31r*^*1/Df*^	84	50 (60)	22.5	0.09	349.8	22.9	5
*Pdfr*^*5304*^; *Dh31*^*#51*^	88	17 (19)	23.4	0.15	320.2	29.9	5

Free-running rhythms were calculated from locomotor activity data sets from DD1 to DD10 for each genotype. Figures associated with the free-running rhythm data in each genotype are shown.

We also determined that the free-running period of the *Dh31*^*#51*^*;Pdf*^*01*^ double mutants was 23.1 h (Fig. 1I and Table 1), which was slightly longer than that of the *Pdf*^*01*^ mutants (22.5 h; Fig. 1H and Table 1) and shorter than that of the *Dh31*^*#51*^ mutants (24.4 h; Fig. 1G and Table 1). These data indicate that the *Dh31* mutation did not enhance the shorter period caused by the *Pdf* mutation.

In terms of morning anticipation, compared to WT flies, both the *Dh31*^*#51*^*;Pdf*^*01*^ double mutants and *Dh31*^*#51*^ mutants exhibited abnormal morning anticipation (Fig. S1 and Table S1). However, the anticipation indexes of the *Dh31*^*#51*^*;Pdf*^*01*^ double mutants and *Dh31*^*#51*^ mutants were not significantly different (Table S2). Furthermore, both the *Dh31*^*#51*^*;Pdf*^*01*^ double mutants and *Pdf* ^*01*^ mutants similarly exhibited approximately one-hour advanced peaks in evening activity, which occurred at ZT 10.5–11 (Figs 1D,E and S1 and Tables S3, S4). Thus, the *Dh31* mutation did not enhance the abnormal patterns of morning and evening anticipation caused by the *Pdf* mutation.

In summary, we determined that the lack of DH31 strongly enhanced the arrhythmic phenotype induced by the *Pdf* mutation but did not affect the free-running period, morning anticipation or timing of the evening activity peak in *Dh31*^*#51*^*;Pdf*^*01*^ double mutants. Therefore, we examined the functions of DH31 in regulating free-running rhythmicity, with a particular focused on the relationship between DH31 and PDF.

### DH31 in *tim-Gal4*-expressing neurons contributes to free-running rhythmicity

Given that *Dh31*^*#51*^*;Pdf*^*01*^ double-mutant flies exhibited disrupted free-running rhythmicity and that *Dh31*^*#51*^ mutants still showed normal free-running rhythmicity (Fig. 1), it is likely that an abnormal *Dh31*^*#51*^ phenotype might be invisible in the presence of normal PDF signaling. To examine this possibility, we asked whether DH31 expression in clock neurons could overcome the changes in rhythmicity identified in *Dh31*^*#51*^*;Pdf*^*01*^ double mutants.

Because we and others showed that DH31 is expressed in a subset of circadian clock cells (DN1ps)^14,15^, we first expressed DH31 in DN1ps using *R18H11-Gal4* (a DN1p driver) (Fig. S4B). However, 73% of the rescued flies exhibited arrhythmicity (Figs S3G, S4B, and Table 1: *R18H11-Gal4* > *UAS-Dh31*, *Dh31*^*#51*^*;Pdf*^*01*^), which was similar to the results for the *Gal4 and UAS* control flies (Fig. S3D,F and Table 1: *R18H11-Gal4*/+, *Dh31*^*#51*^*;Pdf*^*01*^, +*/UAS-Dh31*, *Dh31*^*#51*^*;Pdf*^*01*^). These data suggest that DH31 expression in *R18H11-Gal4-*expressing neurons is not sufficient to restore free-running rhythmicity.

**Figure 2:**
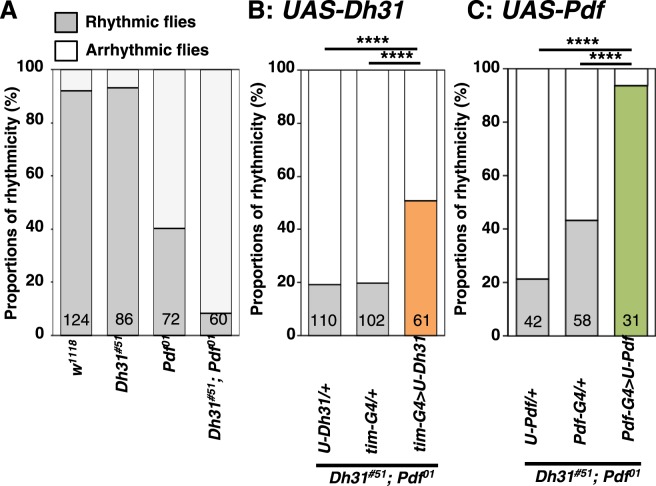
DH31 expression in *tim-Gal4-*expressing neurons or PDF expression in LNvs rescued severe arrhythmicity in *Dh31*-*Pdf* double mutants. (**A-C**) Comparison of free-running rhythms in different genotypes: *w*^*1118*^, *Dh31*^*#51*^, *Pdf*^*01*^ and *Dh31*^*#51*^*;Pdf*^*01*^ **(A**), *UAS-Dh31*/+, *tim-Gal4*/+ and *tim-Gal4* > *UAS-Dh31* from the *Dh31*^*#51*^*;Pdf*^*01*^ double-mutant background **(B)** and *UAS-Pdf* /+, *Pdf-Gal4*/+ and *Pdf-Gal4* > *UAS-Pdf* from the *Dh31*^*#51*^*;Pdf*^*01*^ double-mutant background **(C)**. The data in Fig. 2A are the reproduced from Fig. 1A. The proportions of rhythmic (gray bar) and arrhythmic (white bar) flies over 10 days in DD were compared via χ^2^ analysis. ****P < 0.0001. Numbers in the bar graphs represent the number of flies.

*R18H11-Gal4* is expressed in only ~ four to six DN1ps^14^, and we also found that DH31 is expressed in anterior DN1s (DN1as) (Supplemental Fig. S5). As such, DH31 expression from non-*R18H11-Gal4-*expressing DN1s might also play a role in regulating free-running rhythmicity. We therefore expressed DH31 using *tim-Gal4* (a driver for all clock cells) in the *Dh31*^*#51*^*;Pdf*^*01*^ double mutants and assessed whether DH31 expression alone could prevent the severe arrhythmicity. We determined that 51% of the rescued flies showed restored free-running rhythmicity (Figs 2B and S3P, and Table 1: *tim-Gal4* > *UAS-Dh31*, *Dh31*^*#51*^*;Pdf*^*01*^), while the *Gal4* and *UAS* control flies from the double-mutant background still exhibited severe arrhythmic phenotypes (Figs 2B and S3B,F, and Table 1: *tim-Gal4*/+, *Dh31*^*#51*^; *Pdf*^*01*^, +*/UAS-Dh31*, *Dh31*^*#51*^; *Pdf*^*01*^). The data indicated that DH31 expression in *tim-Gal4*-expressing neurons in *Dh31*^*#51*^*;Pdf*^*01*^ mutants is sufficient to recover a similar level of rhythmicity to that of *Pdf*^*01*^ mutants (Fig. 2A,B) and suggested that DH31 in *tim-Gal4*-expressing neurons contributes to free-running rhythmicity.

### PDF and DH31 regulate free-running rhythmicity in a hierarchical fashion

Given that *Pdf*^*01*^ single mutants exhibited an arrhythmic phenotype, PDF should be able to regulate a free-running rhythm without DH31. To confirm this hypothesis, PDF was expressed in LNvs using *Pdf-Gal4* (a LNv driver) in *Dh31*^*#51*^*;Pdf*^*01*^ double-mutant flies. These flies strongly recovered their free-running rhythm, with a rhythmicity level that was similar to that of *Dh31*^*#51*^ and WT flies (Figs 2A,C and S3O, and Table 1: *Pdf-Gal4* > *UAS-Pdf*, *Dh31*^*#51*^*;Pdf*^*01*^), suggesting that PDF secretion from LNvs is sufficient to restore rhythmicity in these double-mutant flies. Taken together, we concluded that PDF and DH31 regulate free-running rhythmicity in a hierarchical fashion, in which PDF functions in a primary role, and DH31 functions in a secondary role.

Importantly, the disruption of morning anticipation and the advanced peak in evening activity in *Dh31*^*#51*^*;Pdf*^*01*^ double mutants were also recovered in *Pdf-Gal4* > *UAS-Pdf*, *Dh31*^*#51*^*;Pdf*^*01*^ flies (S2I,L,M and Tables S1 and S2). These data also highlight that the overexpression of PDF from LNvs is sufficient to restore normal phenotypes in the double mutants and to prevent the abnormal morning anticipation phenotype in *Dh31* mutants.

### Both DH31 and PDF act on DN1ps to regulate free-running rhythmicity

**Figure 3:**
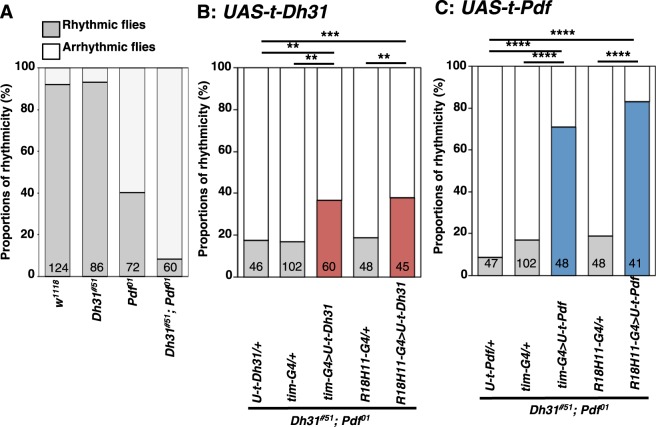
t-DH31 or t-PDF expression in DN1ps prevented severe arrhythmicity in *Dh31*-*Pdf* double mutants. (**A-C**) Comparison of free-running rhythms in different genotypes: *w*^*1118*^, *Dh31*^*#51*^, *Pdf*^*01*^ and *Dh31*^*#51*^*;Pdf*^*01*^ **(A)**, *UAS-t-Dh31*/+, *tim-Gal4*/+, *tim-Gal4* > *UAS-t-Dh31*, *R18H11-Gal4 (DN1ps driver)*/+ and *R18H11-Gal4* > *UAS-t-Dh31* from the *Dh31*^*#51*^*;Pdf*^*01*^ double-mutant background **(B)** and *UAS-t-Pdf* /+, *tim-Gal4*/+, *tim-Gal4* > *UAS-t-Pdf*, *R18H11-Gal4*/+ and *R18H11-Gal4* > *UAS-t-Pdf* from the *Dh31*^*#51*^*;Pdf*^*01*^ double-mutant background **(C)**. The data in Fig. 3A are reproduced from Fig. 1A. The proportions of rhythmic (gray bar) and arrhythmic (white bar) flies over 10 days in DD were compared via χ^2^ analysis. ****P < 0.0001, ***P < 0.001 and **P < 0.01. Numbers in the bar graphs represent the number of flies.

To determine how PDF and DH31 regulate free-running rhythmicity at the cellular level, we first verified that both PDF and DH31 act on clock cells to regulate free-running rhythmicity. Membrane-tethered peptides have both linker and anchor peptides that couple with the cell membrane, which results in cell-autonomous binding and the constant activation of the receptors on specific cells^17,18^. By using tethered-PDF (t-PDF), we determined that t-PDF expression in all clock cells using *tim-Gal4* restored rhythmicity to 71% in the double mutants, showing a rhythmicity close to that of the *Dh31*^*#51*^ flies (Figs 3A,C and S3B,H,I, and Table 1: *tim-Gal4* > *UAS-t-Pdf*, *Dh31*^*#51*^*;Pdf*^*01*^). Similarly, t-DH31 expression in all clock cells using *tim-Gal4* also restored rhythmicity to 37%, which was similar to the rhythmicity of the *Pdf*^*01*^ flies (Figs 3A,B and S3A–C, and Table 1: *tim-Gal4* > *UAS-t-Dh31*, *Dh31*^*#51*^*;Pdf*^*01*^). Therefore, we confirmed that both PDF and DH31 act on clock cells to regulate free-running rhythmicity.

LNvs are the main clock cells that regulate locomotor activity rhythms^2,3,5^, and bath application of PDF or DH31 can activate LNvs^19^. Therefore, we assessed whether DH31 and PDF act on LNvs to regulate rhythmicity. However, neither t-DH31 nor t-PDF expression in LNvs using *Pdf-Gal4* was able to restore rhythmicity compared to the phenotypes of the Gal4 control flies (Figs S3K,L,M and S4C,D and Table 1: *Pdf-Gal4* > *UAS-t-Dh31* and *Dh31*^*#51*^*;Pdf*^*01*^, *Pdf-Gal4* > *UAS-t-Pdf*, *Dh31*^*#51*^*;Pdf*^*01*^). Therefore, our data suggest that both PDF and DH31 are less likely to act on LNvs to modulate rhythmicity.

We subsequently focused on DN1s because small LNvs (sLNvs) project to DN1s^20–22^, PDF acts on DN1ps to regulate locomotor activity^22,23^, and *Pdfr* expression in DN1ps restores the dampened free-running rhythm in *Pdfr* mutant flies^23^. We determined that flies with t-PDF expression in DN1ps using *R18H11-Gal4* showed 83% rhythmicity, which was close to the rhythmicity of the *Dh31*^*#51*^ flies (Figs 3A,C and S3J, and Table 1: *R18H11-Gal4* > *UAS-t-Pdf*, *Dh31*^*#51*^*;Pdf*^*01*^). Therefore, we confirmed that t-PDF expression in DN1ps rescues the severe disruption of free-running rhythm identified in *Dh31*^*#51*^*;Pdf*^*01*^ double mutants.

To determine whether DH31 also acts on the same group of clock cells, we expressed t-DH31 in DN1ps and assessed the effect on free-running rhythmicity. When t-DH31 was expressed in the DN1ps of *Dh31*^*#51*^*;Pdf*^*01*^ double-mutant flies using the *R18H11-Gal4* driver, the free-running rhythmicity was restored to the same level as that of the *Pdf*^*01*^ flies (38%, Figs 3A,B and S3E, and Table 1: *R18H11-Gal4* > *UAS-t-Dh31*, *Dh31*^*#51*^*;Pdf*^*01*^). The rhythmicity of these flies was significantly different from that of the *Gal4* or *UAS* control flies from the double-mutant background (Fig. 3B and Table 1). These results suggest that DH31 also acts on DN1ps to modulate free-running rhythmicity. Thus, our data suggest that both PDF and DH31 can act on DN1ps to regulate free-running rhythmicity.

### *Dh31* mutation did not enhance the abnormal molecular oscillations caused by the *Pdf* mutation

To further examine the role of DH31 in the arrhythmic phenotype, we focused on the molecular oscillations in each clock cell. Given that the *Pdf* mutation causes abnormal molecular oscillations in clock cells^8,9^ and that the *Dh31* mutation enhanced the abnormal free-running rhythms caused by the *Pdf* mutation (Fig. 1A), we suspected that molecular oscillations in the clock cells of *Dh31*^*#51*^*;Pdf*^*01*^ double mutants would be severely dampened compared to those of *Pdf*^*01*^ mutants.

**Figure 4:**
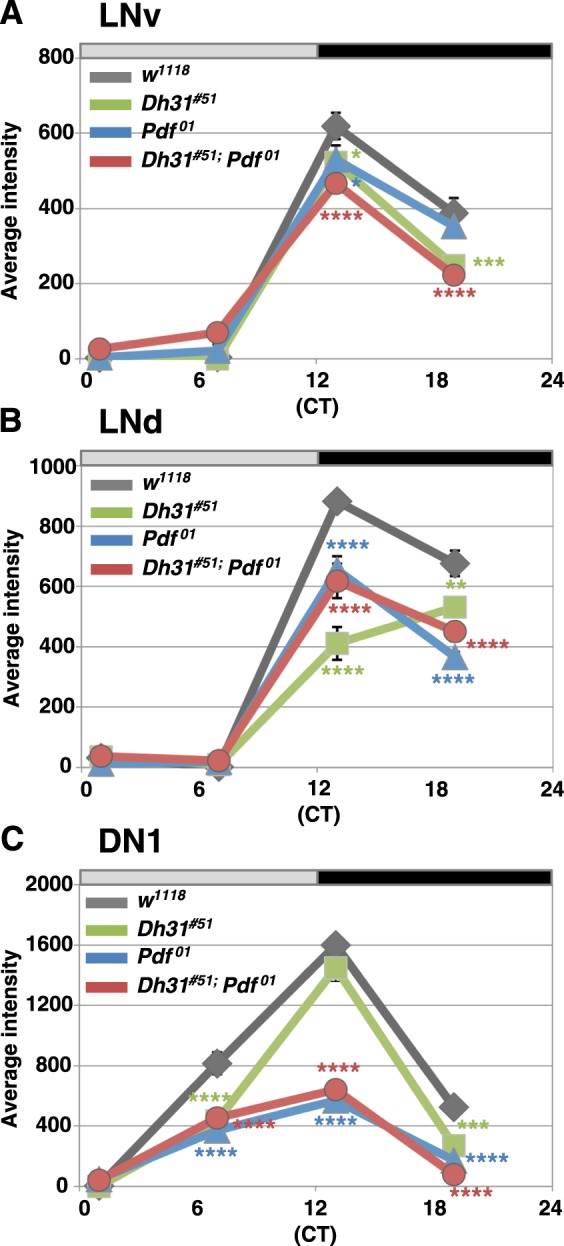
*Dh31* mutation did not enhance the abnormal molecular oscillations caused by *Pdf* mutation. The average levels of VRI expression in 10 brain hemispheres in each subgroup of clock cells (LNv, LNd and DN1) among WT, *Dh31*^*#51*^, *Pdf*^*01*^ and *Dh31*^*#51*^*;Pdf*^*01*^ double mutants at the indicated time points ((circadian time (CT) 1, 7, 13 and 19) in DD3. LNv (**A**), LNd (**B**) and DN1 (**C**). The detailed data of the average intensity are shown in Table S5 The variation of the average intensity in a day in each genotype was compared using two-way ANOVA and Sidak’s multiple-comparison test. The results for comparisons with WT flies at each time point are shown: ****P < 0.0001, ***P < 0.001, **P < 0.01 or *P < 0.05. The remaining comparisons are shown in Table S4.

To test this possibility, we examined the molecular oscillations in each group of clock cells (LNv, LNd, and DN1) by measuring the expression of Vrille (VRI), which is a component of the second clock feedback loop in the core molecular clock system^24^. In most cases, severe arrhythmicity in the *Dh31*^*#51*^*;Pdf*^*01*^ double mutants was identified by three days after the shift from LD to DD; thus, we compared the expression levels of VRI in each mutant maintained at DD3 (Fig. S3Q). We determined that the intensities of VRI expression in LNvs and LNds in all mutant flies (*Dh31*^*#51*^, *Pdf*^*01*^ and *Dh31*^*#51*^*;Pdf*^*01*^) were less than those in WT flies at ZT 13 and 19 (Figs 4A,B and S6 and Tables S5, S6). Notably, the peak of VRI expression in LNds in *Dh31*^*#51*^ was delayed compared to that in WT and the other mutants (Figs 4B and S6), suggesting that DH31 is involved in regulating molecular rhythms in LNds. Furthermore, the *Pdf*^*01*^ and *Dh31*^*#51*^*;Pdf*^*01*^ mutants exhibited a severe disruption in the molecular rhythm in their DN1s (Figs 4C and S6 and Tables S5, S6). However, there was no significant difference between the *Pdf*^*01*^ and *Dh31*^*#51*^*;Pdf*^*01*^ mutants in LNvs, LNds or DN1s (Figs 4 and S6 and Tables S5, S6). Therefore, the data did not show a significant difference in VRI expression between *Pdf*^*01*^ and *Dh31*^*#51*^*;Pdf*^*01*^ double mutants, suggesting that the *Dh31* mutation does not enhance the abnormal molecular oscillations caused by the *Pdf* mutation. Thus, the role of *Dh31* in regulating free-running rhythmicity may differ from that of *Pdf*.

### *Dh31r* mutation did not enhance the severe disruption of locomotor activity rhythms in *Pdfr* mutant flies

**Figure 5:**
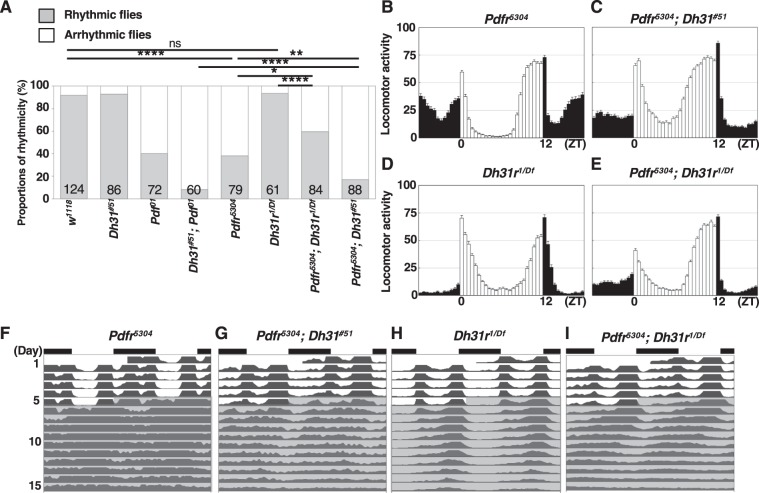
DH31R is not involved in locomotor activity. (**A**) Comparison of free-running rhythms in different genotypes. The proportions of rhythmic (gray bar) and arrhythmic (white bar) flies over 10 days in DD were compared via χ^2^ analysis. ****P < 0.0001, **P < 0.01 or *P < 0.05. Numbers in the bar graph represent the number of flies. (**B-E**) Average daily actogram over 4 days in LD for each genotype: *Pdfr*^*5304*^ (**B**), *Pdfr*^*5304*^; *Dh31*^*#51*^ (**C**), *Dh31r*^*1/Df*^ (**D**) and *Pdfr*^*5304*^*;Dh31*^*1/Df*^ (**E**). (**F-I**) Double-plotted averaged actogram of rhythmic flies over 5 days in LD and 10 days in DD for each genotype: *Pdfr*^*5304*^ (**F**), *Pdfr*^*5304*^*;Dh31*^*#51*^ (**G**), *Dh31r*^*1/Df*^ (**H**) and *Pdfr*^*5304*^*;Dh31*^*1/Df*^ (**I**).

We subsequently focused on the functions of PDF and DH31 receptors in locomotor activity rhythms. In *Dh31 receptor* (*Dh31r*) *loss-of-function* mutant flies (*Dh31r*^*f05546*^*/Df(2 R)BSC273*, referred to here as *Dh31r*^1^*/*^*Df*^), *Dh31r* mRNA levels in the head were 38% of those observed in *w*^*1118*^ flies^25^. We recently found that these mutant flies showed abnormal TPRs; however, they exhibited normal locomotor activity rhythms (Fig. 5A,D,H and Table 1: *Dh31r*^*1/Df*^)^25^. Given the phenotype of *Dh31*^*#51*^*;Pdf*^*01*^ double mutants in locomotor activity rhythms, we expected the *Dh31r* mutation to also enhance the *Pdfr* mutant phenotype. To this end, we created *Pdfr*^*5304*^*;Dh31r*^*1/Df*^ double mutants. First, we confirmed that *Pdfr*^*5304*^ mutants exhibited a weak rhythmicity (51% rhythmic and power = 323.6), shorter period (23.3 h), loss of morning anticipation and advanced phases in the evening activity peak (Fig. 5A,B,F and Tables 1 and S1), which were very similar to the phenotype of *Pdf*^*01*^ mutants. These findings suggest that *Pdfr*^*5304*^ mutants represent a phenocopy of *Pdf*^*01*^ mutants^12,26,27^. However, the *Pdfr*^*5304*^*;Dh31r*^*1/Df*^ double-mutant phenotype still exhibited 60% rhythmicity (Fig. 5A,I and Table 1), which was similar to that of the *Pdfr*^*5304*^ single mutants (51% rhythmic, Table 1). The morning anticipation and phase-advanced evening activity peak phenotypes were also not influenced by the double mutation of *Pdfr* and *Dh31r* (Fig. 5E and Tables S1 and S2). Thus, these data indicate that the *Dh31r* mutation did not enhance the arrhythmic phenotype caused by the *Pdfr* mutation.

Furthermore, as a control, we generated *Pdfr*^*5304*^*;Dh31*^*#51*^ double mutant flies and tested their locomotor activity rhythms. *Pdfr*^*5304*^*;Dh31*^*#51*^ double mutant flies showed only 23% rhythmicity in free running (Fig. 5A,G and Table 1), which was significantly lower than that of *Pdfr*^*5304*^ or *Pdfr*^*5304*^*;Dh31r*^*1/Df*^ double-mutant flies (51% or 60%, respectively) (Fig. 5A,F,I and Table 1). The results suggested that both PDF and DH31 signals regulate robust locomotor activity rhythms but that DH31R is unlikely to regulate locomotor activity rhythms.

## Discussion

### PDF and DH31 regulate free-running rhythmicity in a hierarchical fashion in DN1ps

**Figure 6:**
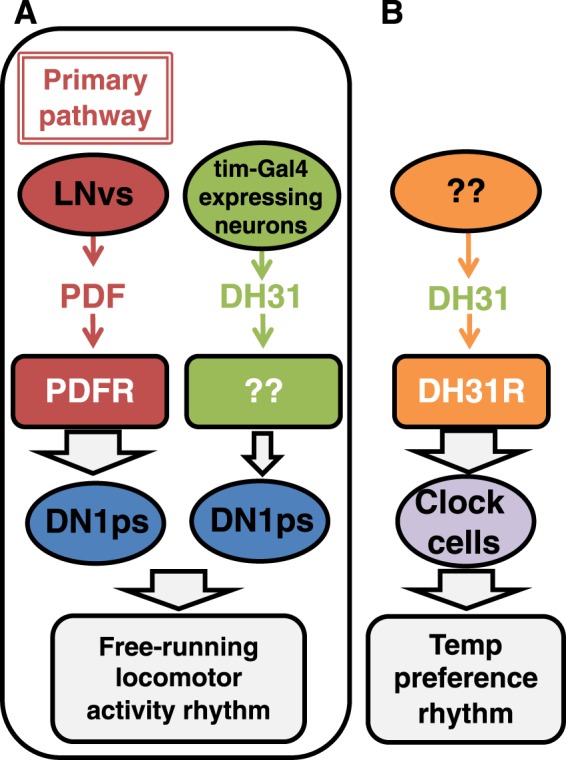
Schematic diagram of the relationship between DH31 and PDF in regulating free-running rhythmicity. (**A**) A model of the relationship between DH31 and PDF in regulating free-running rhythms. In the primary pathway, PDF acts on DN1ps via the PDFR. In the secondary pathway, DH31 acts on DN1ps via unknown receptor(s). PDF and DH31 independently regulate free-running rhythmicity; however, the effect of the primary pathway on rhythmicity is dominant to that of the secondary pathway. (**B**) We have previously shown that DH31R regulates TPR^25^.

We demonstrated a novel function of DH31 in regulating *Drosophila* locomotor activity rhythms. We showed that *Dh31*^*#51*^ mutants maintained a robust free-running rhythm (Fig. 1 and Table 1), whereas *Dh31*^*#51*^*;Pdf*^*01*^ double-mutant flies exhibited a severe disruption of their free-running rhythm compared to *Pdf*^*01*^ mutants (Fig. 1 and Table 1). These findings suggest that *Dh31*^*#51*^ mutants maintain a robust free-running rhythm because the primary factor, PDF, can sustain a strong rhythm (Figs 1 and 6). We showed that ~40% of *Pdf*^*01*^ single-mutant flies exhibited a preserved rhythmic state, which is because DH31 can partially support free-running rhythmicity. Thus, the severe disruptions of free-running rhythm in *Pdf*^*01*^ and *Dh31*^*#51*^ double-mutant flies is likely caused by the loss of both pathways.

PDF is secreted from the main circadian neurons, LNvs, and acts on other clock cells through PDFR to synchronize and maintain robust molecular rhythms^11,19^. We showed that PDF expression from LNvs in *Dh31*^*#51*^*;Pdf*^*01*^ mutants restored rhythmicity (Fig. 2C), in contrast to t-PDF expression in LNvs (Fig. S4D), indicating that an autoreceptor of PDF signals in LNvs is not sufficient to maintain rhythmicity. Instead, we showed that t-PDF expression in DN1ps restored rhythmicity, which suggests that PDF signaling in DN1ps is sufficient to maintain robust free-running rhythmicity (Fig. 3C). Recently, the responsiveness to PDF was shown to be strongly altered for 24 h via RalA GTPase in sLNvs^28^. Therefore, we expect that the continuous activation of PDFR by t-PDF generates rhythmic downstream signaling in PDFR-expressing neurons.

We also showed that molecular oscillations in DN1s were strongly dampened in *Pdf*^*01*^ mutants compared with WT flies (Fig. 4C). These data are consistent with previous studies in which the molecular oscillations of PER in *Pdf*^*01*^ mutants held under DD conditions were dampened in DN1s^10^ and the genetic manipulation of the circadian clocks in PDF-positive cells altered the molecular rhythms in DN1ps^23,29^. Furthermore, *Pdfr* expression in DN1ps has been reported to prevent the arrhythmic phenotype in *Pdfr* ^5304^ mutants^23^. These findings support the idea that PDF is secreted from LNvs and acts on DN1ps to regulate free-running rhythmicity.

Furthermore, we showed that t-DH31 expression in DN1ps rescued the *Pdf*^*01*^ and *Dh31*^*#51*^ double-mutant phenotypes, which suggests that DH31 acts on DN1ps to regulate rhythmicity (Fig. 3B). Although it has been suggested that DH31 release might increase at dawn^14^ and that DH31-mRNA expression levels oscillate for 24 h^13^, how t-DH31 expression causes rhythmic behavioral output remains unclear. Because DH31 can modestly activate PDFR *in vitro*^12^, we cannot exclude the possibility that t-DH31 overexpression might simply activate PDFR in DN1ps instead of the intrinsic PDF signals, thereby restoring locomotor activity rhythms in the flies. However, the rhythmicity of *Dh31*^*#51*^*;Pdf*^*01*^ mutants overexpressing t-DH31 in *tim-Gal4-*expressing neurons or *R18H11-Gal4*-expressing DN1ps only reached levels similar to that of the *Pdf*^*01*^ single-mutant flies (Fig. 3A,B). Therefore, DH31 likely acts on DN1ps separately from the PDF pathway.

Although we and others have shown that DH31 is expressed in a subset of DN1ps^14,15^, DH31 expression using *R18H11-Gal4* did not rescue the *Pdf*^*01*^ and *Dh31*^*#51*^ double-mutant phenotypes (Fig. S4B), suggesting that DH31 expression in *R18H11-Gal4-*expressing neurons is insufficient to maintain rhythmicity. Instead, we showed that DH31 is expressed in DN1as (Fig. S5) and that DH31 expression in *tim-Gal4*-expressing neurons rescued the phenotype (Fig. 2B), which suggests that DH31 expression in clock neurons maintains rhythmicity. That said, given that DH31 is expressed in nonclock neurons^14,15^ and that *tim-Gal4* is expressed in nonclock cells^30^, we cannot exclude the possibility that DH31 expression in nonclock neurons might play a role in rescuing the severe phenotype of *Dh31*^*#51*^*;Pdf*^*01*^ mutants. Alternatively, although DH31 expression in LNvs was not detectable via anti-DH31 antibody staining^15^, a recent RNA-seq analysis detected *Dh31* gene expression in both LNvs and DN1s^13^. Therefore, DH31 expression from LNvs may potentially act on DN1s to support locomotor activity rhythms.

In summary, we propose that PDF and DH31 regulate free-running rhythms in a hierarchical fashion in DN1ps (Fig. 6). As t-DH31 or t-PDF expression in DN1ps resulted in a similar level of rhythmicity as that observed in flies expressing t-DH31 or t-PDF, respectively, in *tim-Gal4-*expressing neurons (Fig. 3), DN1ps are at least one of the important clock cells that regulate free-running rhythmicity.

### The roles of DH31 in locomotor activity rhythms

Given that *Dh31*^*#51*^*;Pdf*^*01*^ mutants exhibited severe arrhythmicity in free-running rhythm, we speculated that the severe arrhythmic phenotype might be a result of abnormal molecular oscillations. However, the molecular oscillations of *Dh31*^*#51*^*;Pdf*^*01*^ mutants were similar to those of *Pdf*^*01*^ mutants (Fig. 4). Therefore, the molecular mechanisms by which DH31 regulates free-running rhythms still remain unclear. Importantly, the peak of VRI expression in LNds in *Dh31*^*#5*^ was at ZT 19, which was delayed compared with those of WT flies and the other mutants (Fig. 4B). The data suggested that DH31 is involved in the regulation of molecular oscillations in LNds. Because LNds are the evening pacemaker^2,3^, the delayed VRI oscillations in LNds might be associated with the longer period of free-running rhythm in *Dh31*^*#51*^ (24.4 h, Table 1).

Recently, the intracellular calcium rhythms in each clock cell were reported to be nonsynchronous and associated with morning and evening peaks in locomotor activity^4^. DH31 signaling may possibly contribute to the downstream output that controls molecular rhythms in pacemaker processes, such as intracellular calcium rhythms. Given that PDF from sLNvs regulates strong molecular rhythms in DN1ps and generates robust free-running rhythms under constant conditions^10,23,29^, DH31 may help maintain vigorous output signals downstream of the molecular clocks in DN1ps.

### DH31 receptor is unlikely to regulate locomotor activity rhythms

We recently showed that both *Dh31r*^*1/Df*^ mutants and flies undergoing *Dh31r* knockdown in their neurons showed normal rhythmicity in the locomotor activity rhythm^25^. In contrast to *Dh31*^*#51*^*;Pdf*^*01*^ double mutants, *Pdfr*^*5304*^;*Dh31r*^*1/Df*^ double mutants did not enhance the arrhythmicity observed in *Pdfr* single mutants (Fig. 5A,F,I and Table 1), which suggests that DH31R does not complement PDFR function; thus, DH31R does not function as a receptor for DH31 in this context. Given that *Dh31r*^*1/Df*^ flies showed a strong abnormality in the TPR phenotype^25^, it is more likely that DH31R does not play an important role in locomotor activity rhythms. However, *Dh31r*^*1/Df*^ mutants are not null^25^, and we cannot exclude the possibility that a small amount of residual DH31R might drive robust locomotor activity rhythms with the PDF pathway.

Which receptors might function with DH31 to regulate free-running rhythmicity? Given that DH31 can activate PDFR *in vitro*^12^, bath applications of DH31 can activate LNvs via PDFR^19^ and DH31 can function as a ligand of PDFR in TPR at the onset of night^15^, PDFR may function as a receptors for both DH31 and PDF in the regulation of free-running rhythmicity. However, because the arrhythmicity of *Pdfr*^*5304*^ mutants was not as severe as that of *Dh31*^*#51*^*;Pdf*^*01*^ mutants (Fig. 5), PDFR does not appear to act as a receptor for DH31 in this context (Fig. 6).

Both DH31R and PDFR are class II G-protein coupled receptors (GPCRs), which also include Hector and Diuretic hormone 44 receptors 1 and 2 (DH44R1 and DH44R2, respectively)^31,32^. Interestingly, the DH44R1 and DH44R2 ligand DH44 has been implicated in circadian output circuits^21,33^. Therefore, although there is no evidence from *in vitro* or *in vivo* experiments, these receptors might nevertheless function as receptors for DH31 to regulate free-running rhythmicity.

### Orchestration of neuropeptides regulates locomotor activity rhythms in species ranging from flies to mammals

The orchestration of neuropeptides is critical for regulating circadian clock functions in species that range from flies to mammals. In mammals, several neuropeptides, including vasoactive intestinal polypeptide (VIP), arginine vasopressin (AVP) and neuromedin S (NMS), are expressed in the SCN, which is the center for circadian clock control^34,35^. The hierarchy of neuropeptide signaling contributes to circadian function in the SCN^36^. Several recent studies in *Drosophila* have identified the neuropeptides, including ion transport peptide (ITP)^37^, neuropeptide F (NPF)^38^, allatostatin A^39^, short neuropeptide F^40^, leucokinin^33^ and DH44^21^, that regulate locomotor activity and sleep. However, given that DH31 complements the function of PDF in regulating free-running rhythmicity in the same clock cells, DH31 not only serves as one of the neuropeptides that regulates circadian rhythms but also might selectively influence PDF function in the regulation of free-running rhythms. Thus, our findings shed new light on the next steps required to improve our understanding of the core neuropeptide regulatory mechanisms involved in the circadian rhythm.

## Materials and Methods

### Fly lines and the generation of transgenic flies

All flies were raised in 12 h light/dark cycles at 25 °C; Zeitgeber Time (ZT) 0 was lights-on, ZT12 was lights-off. *w*^*1118*^ (RRID:BDSC_3605) flies were used as WT flies. *UAS-Dh31* was a kind gift of Dr. Paul Taghert. Membrane-tethered DH31 (UAS-*t-DH31-ML:B4*) and membrane-tethered PDF (*UAS-t-PDF-ML:M2a*) were used^17^. *yw*; *Pdf*^*01*^(RRID:BDSC_26654) flies, *yw;Dh31*^*#51*^ (FBal0304655) flies^16^ and *Pdfr*^*5304*^ (RRID:BDSC_33068) flies were backcrossed with *w*^*1118*^ flies. *tim-Gal4* (RRID:BDSC_7126) (expressed in all clock neurons), *Pdf-Gal4* (RRID:BDSC_6900) (expressed in LNvs), and *R18H11-Gal4* (RRID:BDSC_48832) (expressed in ~4–6 DN1s)^14,20^ were obtained from the Bloomington *Drosophila* Stock Center (stock #7126, # 6900 and #48832, respectively). All Gal4 driver and UAS reporter flies from the *Dh31*^*#51*^*;Pdf*^*01*^double-mutant background were generated via chromosome recombination with *w;Dh31*^*#51*^*;Pdf*^*01*^ double-mutant flies. *Dh31r*^1^ is a P-element insertion mutant (*PBac{WH}Dh31-R*^*f05546*^) and was obtained from the Exelixis Collection at the Harvard Medical School. *Dh31r*^*Df*^ is a deletion mutant (*Df(2R)BSC273*) (RRID:BDSC_23169) and was obtained from the Bloomington Drosophila Stock Center (stock # 23169).

### Behavioral analysis of locomotor activity

Locomotor activity assays and data analysis were performed as previously described^15,41,42^. Flies were reared under 12 h light/dark (LD) cycles at 25 °C. Male flies (1 to 5 days old) were used in the locomotor activity experiments. A *Drosophila* Activity Monitoring (DAM) system (http://www.trikinetics.com/) was placed in an incubator (MIR-154, Sanyo Scientific, Japan). Lights in the incubator (15-W cool white fluorescent lamps (FL15D, TOSHIBA, Japan)) were connected to an electric timer; the light intensity was approximately 800 lux. Locomotor activity was monitored in 12 h LD cycles for five days and in a constant dark condition for more than ten days at 25 °C. The data were analyzed using Actogram J software^43^. Free-running periods and power values were calculated using a chi-square periodogram^42,44^, and flies with a power value < 100 were defined as arrhythmic. Only data from rhythmic flies were averaged to generate a double-plotted actogram. Morning anticipation index (AI) values were calculated as previously described^22,45,46^. Briefly, AI = (total activity 3 h before lights-on)/(total activity 6 h before lights-on). The AIs of all flies over days 2–5 of the LD cycles were averaged in each genotype. The AIs for different genotypes were compared using Tukey’s multiple-comparison test. The time of evening peaks in all flies over days 2–5 of the LD cycles were averaged in each genotype. The averaged time of evening peaks for different genotypes were compared using Tukey’s multiple-comparison test.

### Anti-VRI immunohistochemistry and signal intensity quantification

The signals from VRI antibody staining are relatively strong and specific^47^. Given that VRI is not degraded by light, we think that VRI staining is easier to handle compared with TIM staining. Therefore, we used VRI to examine the molecular oscillations in each group of clock cells (LNv, LNd, and DN1) in Fig. 4. *w*^*1118*^, *Dh31*^*#51*^, *Pdf*^*01*^, and *Dh31*^*#51*^*;Pdf*^*01*^ flies were raised under 12 h LD cycles at 25 °C. Adult male flies were subsequently entrained to LD cycles for 3 to 4 days and then shifted to constant dark conditions for 3 days (DD3). The flies were fixed at CT 1, 7, 11 or 19 with 4% paraformaldehyde in PBST (PBS plus 0.3% Triton X-100) for 2 h at room temperature, following brain dissection. Immunostaining was performed as previously described^15^. Briefly, 5% normal goat serum in PBST was used for blocking and antibody incubations with guinea pig anti-VRI antibody (1:200)^47^ and donkey anti-guinea pig Alexa Fluor® 647 (RRID:AB_10895029) (1:200, Jackson ImmunoResearch). Mounted brains were scanned using a Zeiss LSM5 Pascal confocal microscope. Images were digitally projected as Z-stacks for immunohistochemical analysis. ImageJ software was used to quantify the intensity of the immunostaining signal in each single cell (LNv, LNd and DN1). After background subtraction, the total intensity of each type of clock cell in a brain hemisphere was determined, and the average intensity of 10 brain hemispheres was calculated in Excel.

### Experimental design and statistical analysis

#### Free-running rhythmicity

The proportions of rhythmic and arrhythmic flies in each genotype were compared using χ^2^ analysis in Prism 7 software, GraphPad Software, Inc. The sample number and proportion of rhythmic flies are shown in Table 1.

#### AI and time of evening peak

The AI and time of evening peak in each genotype was compared using one-way ANOVA and the Tukey-Kramer test in Prism 7 software, GraphPad Software, Inc. The value of each AI or time of evening peak, sample number, SEM and detailed statistical analysis results are shown in Tables S1–S4.

#### Signal intensity quantification for immunohistochemistry

The variation of the average intensity in a day in each genotype was compared using two-way ANOVA and Sidak’s multiple-comparison test in Prism 7 software, GraphPad Software, Inc. The results of comparisons with WT flies at each time point are shown in Fig. 4. The remaining comparisons are shown in Table S6. The detailed data of the average intensity, SEM, and sample number are shown in Table S5.

## Supplementary information

Figure 1–6 and Table 1–6

Table S2

Figure legends

## References

[1] Taghert PH, Nitabach MN (2012). Peptide neuromodulation in invertebrate model systems. Neuron.

[2] Stoleru D, Peng Y, Agosto J, Rosbash M (2004). Coupled oscillators control morning and evening locomotor behaviour of Drosophila. Nature.

[3] Grima B, Chelot E, Xia R, Rouyer F (2004). Morning and evening peaks of activity rely on different clock neurons of the Drosophila brain. Nature.

[4] Liang X, Holy TE, Taghert PH (2016). Synchronous Drosophila circadian pacemakers display nonsynchronous Ca(2)(+) rhythms *in vivo*. Science.

[5] Yao Z, Shafer OT (2014). The Drosophila circadian clock is a variably coupled network of multiple peptidergic units. Science.

[6] Lear BC, Zhang L, Allada R (2009). The neuropeptide PDF acts directly on evening pacemaker neurons to regulate multiple features of circadian behavior. PLoS biology.

[7] Renn SC, Park JH, Rosbash M, Hall JC, Taghert PH (1999). A pdf neuropeptide gene mutation and ablation of PDF neurons each cause severe abnormalities of behavioral circadian rhythms in Drosophila. Cell.

[8] Lin Y, Stormo GD, Taghert PH (2004). The neuropeptide pigment-dispersing factor coordinates pacemaker interactions in the Drosophila circadian system. The Journal of neuroscience: the official journal of the Society for Neuroscience.

[9] Yoshii T (2009). The neuropeptide pigment-dispersing factor adjusts period and phase of Drosophila’s clock. The Journal of neuroscience: the official journal of the Society for Neuroscience.

[10] Klarsfeld A (2004). Novel features of cryptochrome-mediated photoreception in the brain circadian clock of Drosophila. The Journal of neuroscience: the official journal of the Society for Neuroscience.

[11] Peng Y, Stoleru D, Levine JD, Hall JC, Rosbash M (2003). Drosophila free-running rhythms require intercellular communication. PLoS biology.

[12] Mertens I (2005). PDF receptor signaling in Drosophila contributes to both circadian and geotactic behaviors. Neuron.

[13] Abruzzi KC (2017). RNA-seq analysis of Drosophila clock and non-clock neurons reveals neuron-specific cycling and novel candidate neuropeptides. PLoS genetics.

[14] Kunst M (2014). Calcitonin gene-related peptide neurons mediate sleep-specific circadian output in Drosophila. Current biology: CB.

[15] Goda T (2016). Drosophila DH31 Neuropeptide and PDF Receptor Regulate Night-Onset Temperature Preference. The Journal of neuroscience: the official journal of the Society for Neuroscience.

[16] Head LM (2015). The influence of light on temperature preference in Drosophila. Current biology: CB.

[17] Choi C (2009). Cellular dissection of circadian peptide signals with genetically encoded membrane-tethered ligands. Current biology: CB.

[18] Choi C (2012). Autoreceptor control of peptide/neurotransmitter corelease from PDF neurons determines allocation of circadian activity in drosophila. Cell Rep.

[19] Shafer OT (2008). Widespread receptivity to neuropeptide PDF throughout the neuronal circadian clock network of Drosophila revealed by real-time cyclic AMP imaging. Neuron.

[20] Guo F (2016). Circadian neuron feedback controls the Drosophila sleep–activity profile. Nature.

[21] Cavanaugh DJ (2014). Identification of a circadian output circuit for rest:activity rhythms in Drosophila. Cell.

[22] Seluzicki A (2014). Dual PDF signaling pathways reset clocks via TIMELESS and acutely excite target neurons to control circadian behavior. PLoS biology.

[23] Zhang L (2010). DN1(p) circadian neurons coordinate acute light and PDF inputs to produce robust daily behavior in Drosophila. Current biology: CB.

[24] Blau J, Young MW (1999). Cycling vrille expression is required for a functional Drosophila clock. Cell.

[25] Goda T (2018). Calcitonin receptors are ancient modulators for rhythms of preferential temperature in insects and body temperature in mammals. Genes Dev.

[26] Hyun S (2005). Drosophila GPCR Han is a receptor for the circadian clock neuropeptide PDF. Neuron.

[27] Lear BC (2005). A G protein-coupled receptor, groom-of-PDF, is required for PDF neuron action in circadian behavior. Neuron.

[28] Klose M (2016). Functional PDF Signaling in the Drosophila Circadian Neural Circuit Is Gated by Ral A-Dependent Modulation. Neuron.

[29] Yao Z, Bennett AJ, Clem JL, Shafer OT (2016). The Drosophila Clock Neuron Network Features Diverse Coupling Modes and Requires Network-wide Coherence for Robust Circadian Rhythms. Cell Rep.

[30] Kaneko M, Park JH, Cheng Y, Hardin PE, Hall JC (2000). Disruption of synaptic transmission or clock-gene-product oscillations in circadian pacemaker cells of Drosophila cause abnormal behavioral rhythms. J Neurobiol.

[31] Hector CE, Bretz CA, Zhao Y, Johnson EC (2009). Functional differences between two CRF-related diuretic hormone receptors in Drosophila. The Journal of experimental biology.

[32] Hewes RS, Taghert PH (2001). Neuropeptides and neuropeptide receptors in the Drosophila melanogaster genome. Genome Res.

[33] Cavey M, Collins B, Bertet C, Blau J (2016). Circadian rhythms in neuronal activity propagate through output circuits. Nat Neurosci.

[34] Welsh DK, Takahashi JS, Kay SA (2010). Suprachiasmatic nucleus: cell autonomy and network properties. Annu Rev Physiol.

[35] Colwell CS (2011). Linking neural activity and molecular oscillations in the SCN. Nature reviews. Neuroscience.

[36] Maywood ES, Chesham JE, O’Brien JA, Hastings MH (2011). A diversity of paracrine signals sustains molecular circadian cycling in suprachiasmatic nucleus circuits. Proceedings of the National Academy of Sciences of the United States of America.

[37] Hermann-Luibl C, Yoshii T, Senthilan PR, Dircksen H, Helfrich-Forster C (2014). The ion transport peptide is a new functional clock neuropeptide in the fruit fly Drosophila melanogaster. The Journal of neuroscience: the official journal of the Society for Neuroscience.

[38] He C (2013). Regulation of circadian locomotor rhythm by neuropeptide Y-like system in Drosophila melanogaster. Insect Mol Biol.

[39] Chen J (2016). Allatostatin A Signalling in Drosophila Regulates Feeding and Sleep and Is Modulated by PDF. PLoS genetics.

[40] Shang Y (2013). Short neuropeptide F is a sleep-promoting inhibitory modulator. Neuron.

[41] Kaneko H (2012). Circadian Rhythm of Temperature Preference and Its Neural Control in Drosophila. Current biology: CB.

[42] Umezaki Y, Yoshii T, Kawaguchi T, Helfrich-Forster C, Tomioka K (2012). Pigment-dispersing factor is involved in age-dependent rhythm changes in Drosophila melanogaster. Journal of biological rhythms.

[43] Schmid B, Helfrich-Forster C, Yoshii T (2011). A new ImageJ plug-in “ActogramJ” for chronobiological analyses. Journal of biological rhythms.

[44] Sokolove PG, Bushell WN (1978). The chi square periodogram: its utility for analysis of circadian rhythms. Journal of theoretical biology.

[45] Harrisingh MC, Wu Y, Lnenicka GA, Nitabach MN (2007). Intracellular Ca2+ regulates free-running circadian clock oscillation *in vivo*. The Journal of neuroscience: the official journal of the Society for Neuroscience.

[46] Sheeba V, Fogle KJ, Holmes TC (2010). Persistence of morning anticipation behavior and high amplitude morning startle response following functional loss of small ventral lateral neurons in Drosophila. PloS one.

[47] Glossop NR (2003). VRILLE feeds back to control circadian transcription of Clock in the Drosophila circadian oscillator. Neuron.

