# Supplementary material for: Neuropeptides PDF and DH31 hierarchically regulate free-running rhythmicity in Drosophila circadian locomotor activity
Source: Sci Rep. 2019 Jan 29;9:838. doi: 10.1038/s41598-018-37107-3 (PMC6351594; doi:10.1038/s41598-018-37107-3)
Supplement: Supplementary file 1 — Figure 1–6 and Table 1–6 [file 41598_2018_37107_MOESM1_ESM.pdf]

Neuropeptides PDF and DH31 hierarchically regulate free-running rhythmicity in *Drosophila* circadian locomotor activity

Tadahiro Goda[1], Yujiro Umezaki[1], Fay Alwattari[1], Hanna W. Seo[1] and Fumika N. Hamada[1,2,3,‡]

Figure S1. Goda et al.

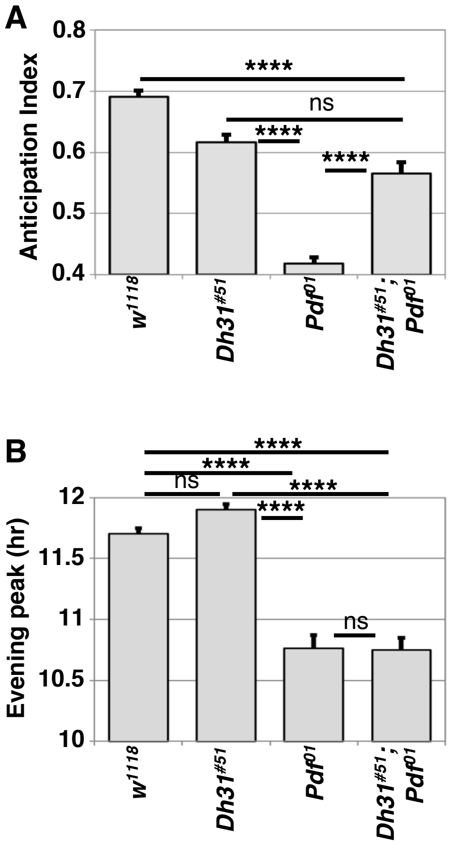

Figure S2. Goda et al.

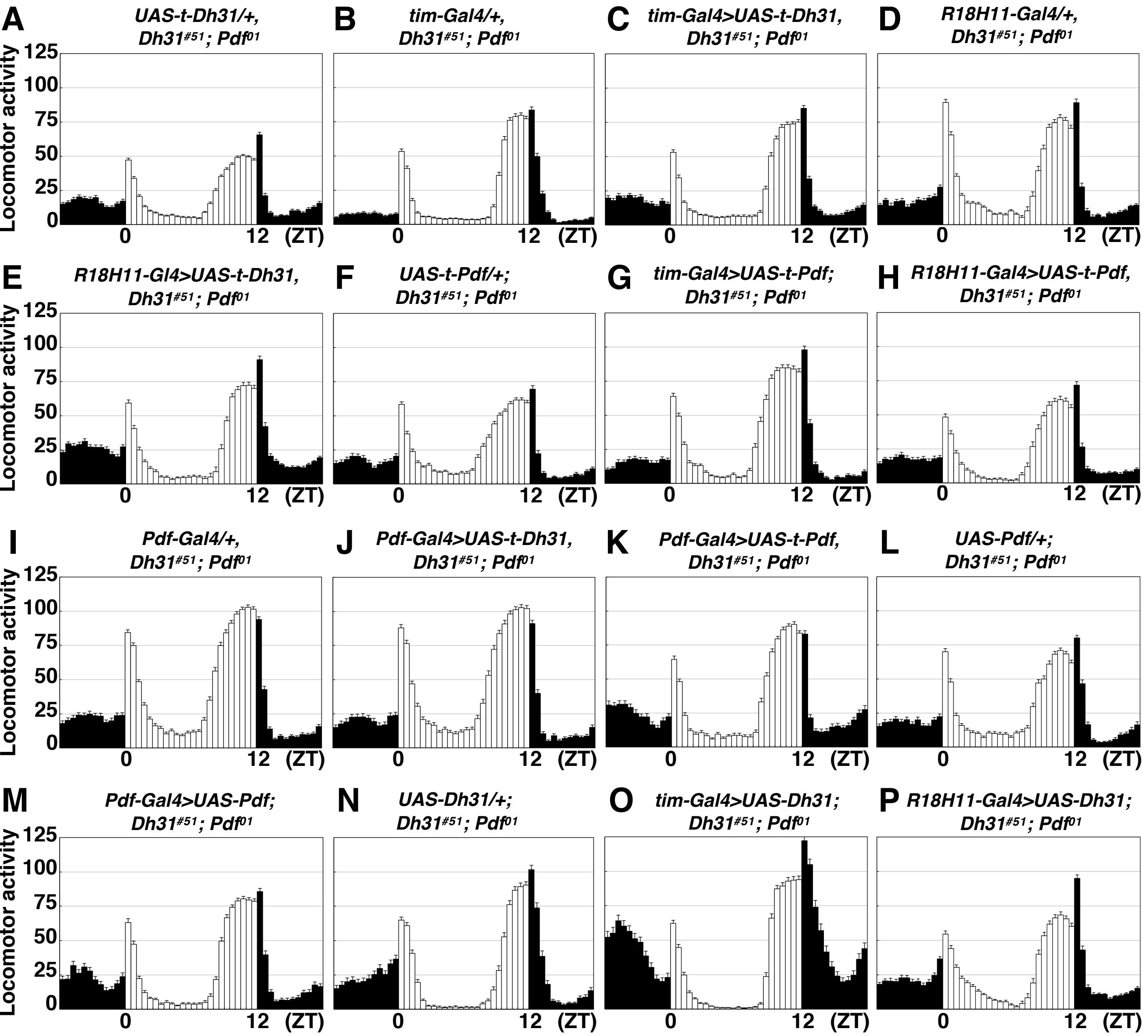

Figure S3. Goda et al.

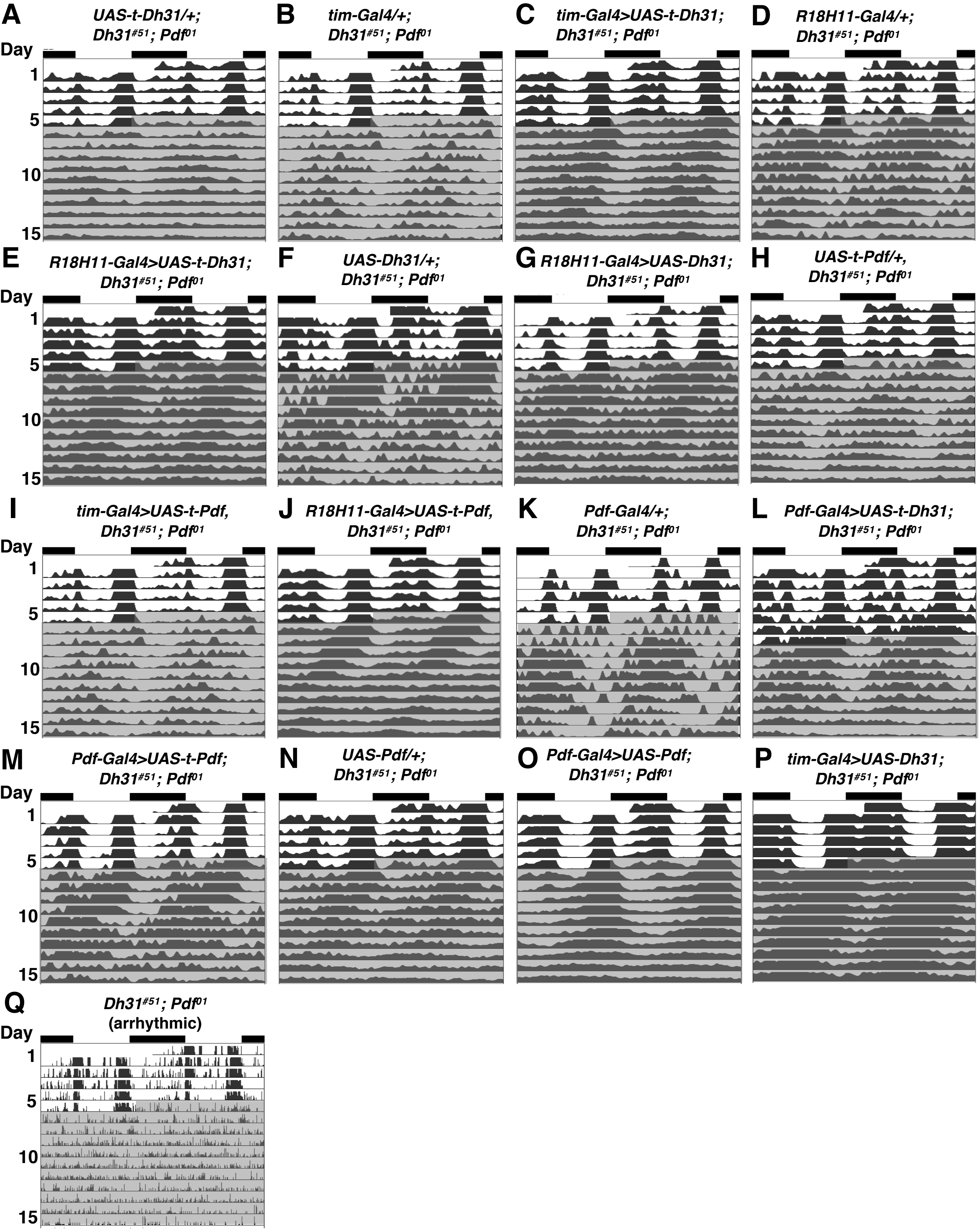

Figure S4. Goda et al.

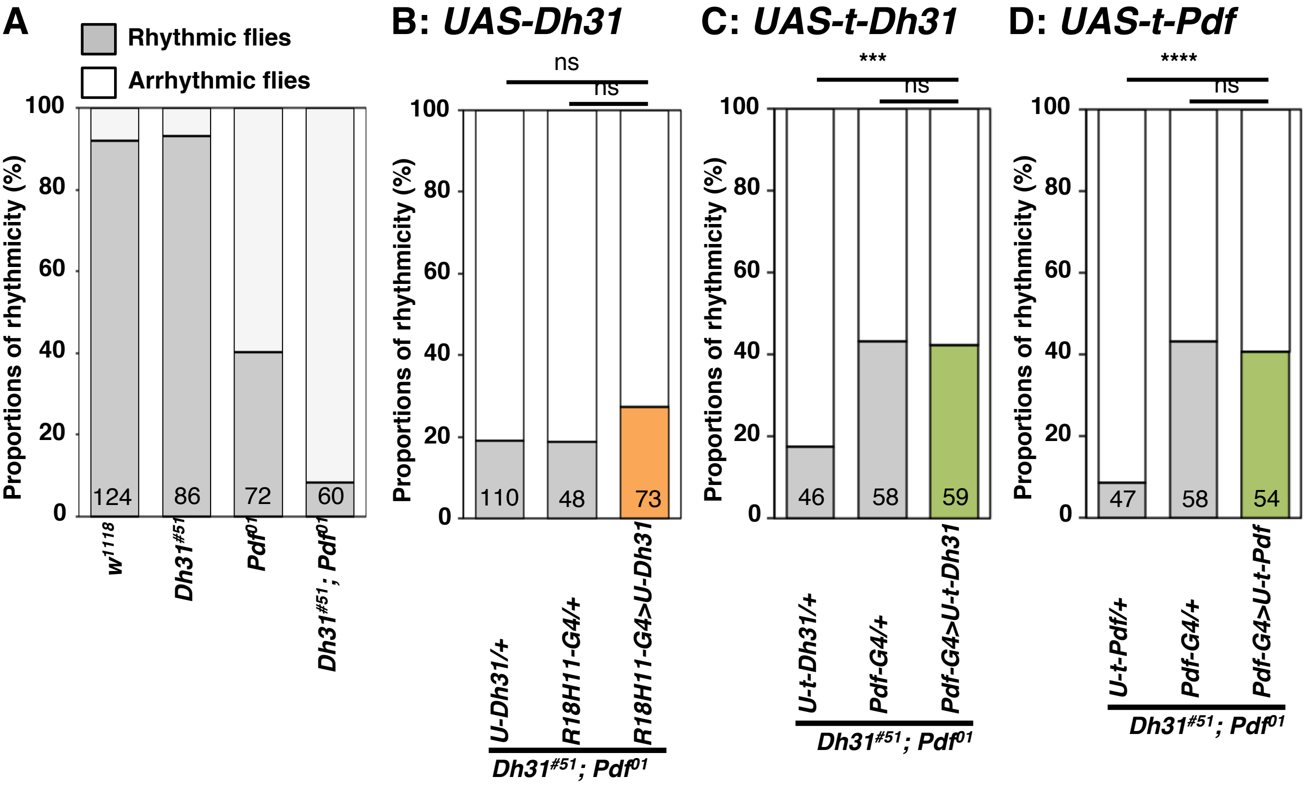

Figure S5. Goda et al.

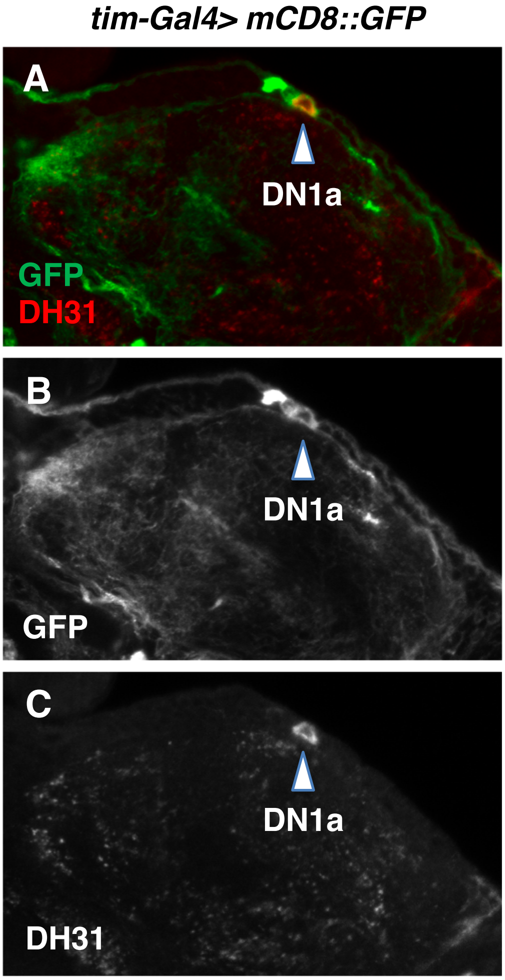

Figure S6. Goda et al.

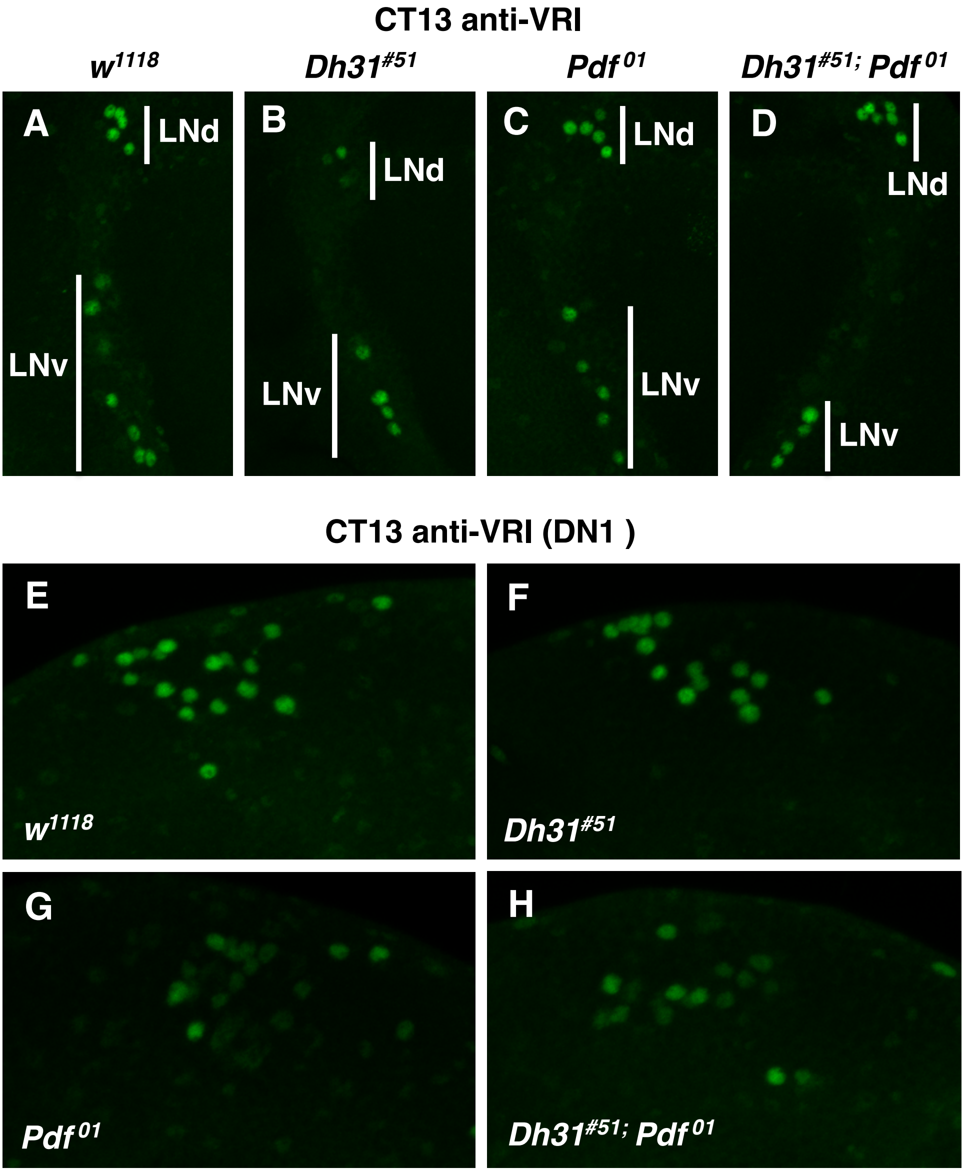

Table. S1. Goda et al.

Morning anticipation index (AI)

Genotype	Average AI	SEM	n
w^{1118}	0.690	0.01	125
Dh31$^{\#51}$	0.616	0.01	85
Pdf^{01}	0.418	0.01	72
Dh31$^{\#51}$*; Pdf*^{01}	0.566	0.02	64
Dh31r$^{1/+}$	0.707	0.03	29
Dh31r$^{Df/+}$	0.745	0.03	32
Dh31r$^{1/Df}$	0.637	0.02	61
Pdfr^{5304}	0.481	0.01	62
Pdfr^{5304}*; Dh31r*$^{1/Df}$	0.588	0.02	94
UAS-Pdf/+, Dh31$^{\#51}$*; Pdf*^{01}	0.531	0.02	48
Pdf-Gal4/+, Dh31$^{\#51}$*; Pdf*^{01}	0.549	0.02	63
Pdf-Gal4>UAS-Pdf, Dh31$^{\#51}$*; Pdf*^{01}	0.635	0.03	33

Table. S2. Goda et al.

One-way ANOVA for anticipation index
(p <0.0001: 1st, 3rd and 5th columns, P = 0.0168: 2nd column, P = 0.0073: 4th column)

Tukey's multiple comparisons test	Mean Diff.	q	P Value	Summary
w^{1118} *vs*. $Dh31^{\#51}$	0.07386	6.213	<0.0001	****
w^{1118} *vs*. Pdf^{01}	0.2719	21.74	<0.0001	****
w^{1118} *vs*. $Dh31^{\#51}$; Pdf^{01}	0.1248	9.599	<0.0001	****
$Dh31^{\#51}$ *vs*. Pdf^{01}	0.1981	14.63	<0.0001	****
$Dh31^{\#51}$ *vs*. $Dh31^{\#51}$; Pdf^{01}	0.0509	3.637	0.0513	ns
Pdf^{01}*vs*. $Dh31^{\#51}$; Pdf^{01}	-0.1472	10.13	<0.0001	****
UAS-Pdf/+, $Dh31^{\#51}$; Pdf^{01} vs. *Pdf-Gal4/+*, $Dh31^{\#51}$; Pdf^{01}	-0.01797	1.55	0.8389	ns
UAS-Pdf/+, $Dh31^{\#51}$; Pdf^{01} vs. *Pdf-Gal4>UAS-Pdf*, $Dh31^{\#51}$; Pdf^{01}	-0.1039	4.941	0.0175	*
Pdf-Gal4/+, $Dh31^{\#51}$; Pdf^{01} vs. *Pdf-Gal4>UAS-Pdf*, $Dh31^{\#51}$; Pdf^{01}	-0.08589	3.5	0.0454	*
w^{1118} vs. $Dh31r^{1/Df}$	0.0534	2.919	0.1241	ns
w^{1118} vs. $Pdfr^{5304}$	0.2096	12.3	<0.0001	****
w^{1118} vs. $Pdfr^{5304}$; $Dh31r^{1/Df}$	0.1027	6.857	<0.0001	****
$Dh31r^{1/Df}$ vs. $Pdfr^{5304}$	0.1562	7.166	<0.0001	****
$Dh31r^{1/Df}$ vs. $Pdfr^{5304}$; $Dh31r^{1/Df}$	0.04927	2.365	0.2165	ns
$Pdfr^{5304}$ vs. $Pdfr^{5304}$; $Dh31r^{1/Df}$	-0.1069	5.957	0.0002	***

Table. S3. Goda et al.

Genotype	Evening peak (hr)	SEM	n
w^{1118}	11.70	0.043	125
Dh31$^{\#51}$	11.90	0.046	85
Pdf^{01}	10.76	0.109	74
Dh31$^{\#51}$; *Pdf*^{01}	10.75	0.100	64

Table. S4. Goda et al.

One-way ANOVA for evening peak
$p < 0.0001$

Tukey's multiple comparisons test	Mean Diff.	q	P Value	Summary
w^{1118} *vs. Dh31*$^{\#51}$	0.196	3.005	0.1472	ns
w^{1118} *vs. Pdf*^{01}	-0.9405	13.82	<0.0001	****
w^{1118} *vs. Dh31*$^{\#51}$*; Pdf*^{01}	-0.954	13.38	<0.0001	****
Dh31$^{\#51}$ *vs. Pdf*^{01}	-1.136	15.41	<0.0001	****
Dh31$^{\#51}$ *vs. Dh31*$^{\#51}$*; Pdf*^{01}	-1.15	14.98	<0.0001	****
Pdf^{01}*vs. Dh31*$^{\#51}$*; Pdf*^{01}	-0.01351	0.1707	0.9994	ns

Table. S5

LNv	w1118			Dh31#51			Pdf01			Dh31#51; pdf01		
	Mean	SEM	n	Mean	SEM	n	Mean	SEM	n	Mean	SEM	n
CT1	2.0919	4.677630602	10	4.1553	3.794843963	10	3.3411	7.47092672	10	25.8402	5.481351611	10
CT7	3.5358	7.906289155	10	0	0	10	22.3814	16.037746	10	70.0525	17.43892529	10
CT13	619.1195	34.94448211	10	522.2545	28.54858327	10	530.6612	36.52420787	10	467.8301	22.88031709	10
CT19	388.0398	40.69089775	10	248.6185	21.50250041	10	353.4682	31.80957288	10	222.2758	18.99575495	10

LNd	w1118			Dh31#51			Pdf01			Dh31#51; pdf01		
	Mean	SEM	n	Mean	SEM	n	Mean	SEM	n	Mean	SEM	n
CT1	31.9175	6.049647157	10	36.1091	13.5453651	10	12.5894	3.116157064	10	37.7383	10.07199956	10
CT7	1.992	4.454247411	10	9.7026	1.730716615	10	18.2338	6.152209308	10	21.7929	11.05985397	10
CT13	881.8779	28.58831118	10	410.8405	54.52507177	10	654.7772	44.6799312	10	617.6216	56.98185882	10
CT19	675.7692	41.90851269	10	531.6189	23.08816531	10	365.9975	17.5305095	10	449.6733	31.81708683	10

DN1	w1118			Dh31#51			Pdf01			Dh31#51; pdf01		
	Mean	SEM	n	Mean	SEM	n	Mean	SEM	n	Mean	SEM	n
CT1	2.4331	5.440576996	10	0	0	10	47.0919	5.440576996	10	40.7312	6.680536115	10
CT7	815.9402	72.58243904	10	435.8252	26.43854751	10	369.7502	42.37102338	10	454.7082	23.04759305	10
CT13	1596.7894	61.55380351	10	1449.8932	90.87092996	10	571.0533	43.23254911	10	641.9943	40.40339406	10
CT19	523.4064	38.6298134	10	271.8957	23.55609558	10	172.7774	16.31070988	10	78.3092	9.009061641	10

Table. S6

LNv

source of variation	F (DFn, DFd)	P value	Summary
Interaction	F (9, 144) = 5.47	P<0.0001	****
Row Factor	F (3, 144) = 496.1	P<0.0001	****
Column Factor	F (3, 144) = 6.24	P=0.0005	***
Sidaks' multiple comparison test	Mean differences	Adjusted P value	Summary
CT13			
Dh31#51 vs. w1118	-96.87	0.0168	*
Pdf01 vs. w1118	-88.46	0.037	*
Dh31#51; pdf01 vs. w1118	-151.3	<0.0001	****
Pdf01 vs. Dh31#51	8.407	>0.9999	ns
Dh31#51; pdf01 vs. Dh31#51	-54.42	0.4319	ns
Dh31#51; pdf01 vs. Pdf01	-62.83	0.2679	ns
CT19			
Dh31#51 vs. w1118	-139.4	0.0001	***
Pdf01 vs. w1118	-34.57	0.8607	ns
Dh31#51; pdf01 vs. w1118	-165.8	<0.0001	****
Pdf01 vs. Dh31#51	104.8	0.0076	**
Dh31#51; pdf01 vs. Dh31#51	-26.34	0.9578	ns
Dh31#51; pdf01 vs. Pdf01	-131.2	0.0004	***

LNd

source of variation	F (DFn, DFd)	P value	Summary
Interaction	F (9, 144) = 14.64	P<0.0001	****
Row Factor	F (3, 144) = 509.7	P<0.0001	****
Column Factor	F (3, 144) = 22.81	P<0.0001	****
Sidaks' multiple comparison test	Mean differences	Adjusted P value	Summary
CT13			
Dh31#51 vs. w1118	-471	<0.0001	****
Pdf01 vs. w1118	-227.1	<0.0001	****
Dh31#51; pdf01 vs. w1118	-264.3	<0.0001	****
Pdf01 vs. Dh31#51	243.9	<0.0001	****
Dh31#51; pdf01 vs. Dh31#51	206.8	<0.0001	****
Dh31#51; pdf01 vs. Pdf01	-37.16	0.932	ns
CT19			
Dh31#51 vs. w1118	-144.2	0.0031	**
Pdf01 vs. w1118	-309.8	<0.0001	****
Dh31#51; pdf01 vs. w1118	-226.1	<0.0001	****
Pdf01 vs. Dh31#51	-165.6	0.0004	***
Dh31#51; pdf01 vs. Dh31#51	-81.95	0.2422	ns
Dh31#51; pdf01 vs. Pdf01	83.68	0.2215	ns

DN1

source of variation	F (DFn, DFd)	P value	Summary
Interaction	F (9, 144) = 37.01	P<0.0001	****
Row Factor	F (3, 144) = 485.9	P<0.0001	****
Column Factor	F (3, 144) = 109.1	P<0.0001	****

Sidaks' multiple comparison test	Mean differences	Adjusted P value	Summary
CT7			
Dh31#51 vs. w1118	-380.1	<0.0001	****
Pdf01 vs. w1118	-446.2	<0.0001	****
Dh31#51; pdf01 vs. w1118	-361.2	<0.0001	****
Pdf01 vs. Dh31#51	-66.08	0.824	ns
Dh31#51; pdf01 vs. Dh31#51	18.88	0.9997	ns
Dh31#51; pdf01 vs. Pdf01	84.96	0.5979	ns
CT13			
Dh31#51 vs. w1118	-146.9	0.067	ns
Pdf01 vs. w1118	-1026	<0.0001	****
Dh31#51; pdf01 vs. w1118	-954.8	<0.0001	****
Pdf01 vs. Dh31#51	-878.8	<0.0001	****
Dh31#51; pdf01 vs. Dh31#51	-807.9	<0.0001	****
Dh31#51; pdf01 vs. Pdf01	70.94	0.7719	ns
CT19			
Dh31#51 vs. w1118	-251.5	0.0001	***
Pdf01 vs. w1118	-350.6	<0.0001	****
Dh31#51; pdf01 vs. w1118	-445.1	<0.0001	****
Pdf01 vs. Dh31#51	-99.12	0.4179	ns
Dh31#51; pdf01 vs. Dh31#51	-193.6	0.0057	**
Dh31#51; pdf01 vs. Pdf01	-94.47	0.4752	ns

LNd in Dh31#51

Tukey's multiple comparisons test	Mean differences	Adjusted P value	Summary
ZT1 vs. ZT7	26.41	<0.0001	****
ZT1 vs. ZT13	-374.7	<0.0001	****
ZT1 vs. ZT19	-495.5	<0.0001	****
ZT7 vs. ZT13	-401.1	<0.0001	****
ZT7 vs. ZT19	-521.9	<0.0001	****
ZT13 vs. ZT19	-120.8	0.038	*

Supplemental data

Figure S1: The morning anticipation index and period of evening peak in each mutant: *w*^{1118}, *Dh31*$^{\#51}$, *Pdf*^{01}, and *Dh31*$^{\#51}$;*Pdf*^{01}.

Comparison of morning anticipation indexes (A) and periods of evening peaks (B) in different genotypes. The results of a one-way ANOVA among the different genotypes are shown ($P < 0.0001$). Tukey-Kramer test results for comparisons of each genotype: ****$P < 0.0001$. The anticipation index in *Pdf*^{01} is significantly lower than that in *Dh31*$^{\#51}$;*Pdf*^{01} because *Pdf*^{01} flies exhibited a relatively higher level of locomotor activity in the middle of the night than during late night (Fig. 1D), which made the anticipation index less than 0.5 (refer to Table S1 and Materials and Methods).

Figure S2: Average daily actogram over 4 days in LD for each genotype.

Locomotor activities from days two to five in LD were averaged and plotted on the bar graph at each time point. DH31 expression in *tim-Gal4*-expressing neurons in the *Dh31* and *Pdf* double-mutant background (*tim-Gal4* > *UAS-Dh31, Dh31*$^{\#51}$;*Pdf*^{01}) caused a high level of locomotor activity during the night (Fig. S2O). Because DH31 functions as a wake-promoting factor [13], it is possible that DH31 expression from *tim-Gal4*-expressing neurons promote a "wake" state in the night that resulted in the high level of nocturnal locomotor activity.

Figure S3: Double-plotted averaged actogram over 5 days in LD and 10 days in DD for each genotype.

Locomotor activities in only rhythmic flies were averaged and plotted on the double-plotted actogram, with the exception of the actogram from a single arrhythmic fly (**Q**).

Figure S4: DH31 expression in DN1ps, as well as t-DH31 or t-PDF expression in LNvs, did not rescue a severe arrhythmic phenotype in *Dh31-Pdf* double mutants.

(**A-D**) Comparison of free-running rhythms in different genotypes: w^{1118}, $Dh31^{\#51}$, Pdf^{01} and $Dh31^{\#51};Pdf^{01}$ (**A**), *UAS-Dh31/+*, *R18H11-Gal4/+* and *R18H11-Gal4 > UAS-Dh31* from the $Dh31^{\#51};Pdf^{01}$ double-mutant background (**B**), *UAS-t-Dh31/+*, *Pdf-Gal4/+*, *Pdf-Gal4 > UAS-t-Dh31* from the $Dh31^{\#51};Pdf^{0}1$ double-mutant background (**C**), and *UAS-t-Pdf /+*, *Pdf-Gal4/+*, and *Pdf-Gal4 > UAS-t-Pdf* from the $Dh31^{\#51};Pdf^{01}$ double-mutant background (**D**). The data in Fig. S4A are reproduced from Fig. 1A. The proportions of rhythmic (gray bar) and arrhythmic (white bar) flies over 10 days in DD were compared via χ^2 analysis. ****P < 0.0001 and ***P < 0.001. Numbers in the bar graphs represent the number of flies.

Figure S5:

Anti-DH31 antibody immunostaining of *tim-Gal4/+; UAS-CD8::GFP/+* fly brains. Arrowhead shows DN1a. GFP (green) and DH31 (red)(A), GFP (B), and DH31 (C) are shown.

Figure S6:

Anti-VRI antibody immunostaining. *w^{1118}* (A, E), *$Dh31^{\#51}$* (B, F), *Pdf^{01}* (C, G), and *$Dh31^{\#51}$;Pdf^{01}* (D, H) flies were prepared at CT13. LNvs, LNds (A-D) and DN1s (E-H) are shown.

Table S1. Morning anticipation index.

Morning anticipation index (AI) of each genotype is shown.

Table S2. Statistical analysis of morning anticipation.

Data on the statistical analysis comparing morning AIs are shown.

Table S3. Period of evening peak.

Period of evening peak in each genotype is shown.

Table S4. Statistical analysis of period of evening peak.

Data on the statistical analysis comparing period of evening peak in each genotype are shown.

Table S5. Detailed data of VRI expression.

Detailed data of the average intensity in anti-VRI immunohistochemistry are shown.

Table S6. Statistical analysis of VRI expression.

Data on the statistical analysis comparing the VRI molecular oscillations presented in Fig. 4 are shown.
