# Supplementary material for: Neuropeptides PDF and DH31 hierarchically regulate free-running rhythmicity in Drosophila circadian locomotor activity
Source: Sci Rep. 2019 Jan 29;9:838. doi: 10.1038/s41598-018-37107-3 (PMC6351594; doi:10.1038/s41598-018-37107-3)
Supplement: Supplementary file 2 — Table S2 [file 41598_2018_37107_MOESM2_ESM.pdf]

Table. S2. Goda et al.

One-way ANOVA for anticipation index
(p <0.0001: 1st and 3rd groups of rows, P = 0.0168: 2nd group of rows)

Tukey's multiple comparisons test	Mean Diff.	q	P Value	Summary
w^{1118} vs. Dh31$^{\#51}$	0.07386	6.213	<0.0001	****
w^{1118} vs. Pdf^{01}	0.2719	21.74	<0.0001	****
w^{1118} vs. Dh31$^{\#51}$; Pdf^{01}	0.1248	9.599	<0.0001	****
Dh31$^{\#51}$ vs. Pdf^{01}	0.1981	14.63	<0.0001	****
Dh31$^{\#51}$ vs. Dh31$^{\#51}$; Pdf^{01}	0.0509	3.637	0.0513	ns
Pdf^{01}vs. Dh31$^{\#51}$; Pdf^{01}	-0.1472	10.13	<0.0001	****
UAS-Pdf/+, Dh31$^{\#51}$; Pdf^{01} vs. *Pdf-Gal4/+, Dh31$^{\#51}$; Pdf^{01}*	-0.01797	1.55	0.8389	ns
UAS-Pdf/+, Dh31$^{\#51}$; Pdf^{01} vs. *Pdf-Gal4>UAS-Pdf, Dh31$^{\#51}$; Pdf^{01}*	-0.1039	4.941	0.0175	*
Pdf-Gal4/+, Dh31$^{\#51}$; Pdf^{01} vs. *Pdf-Gal4>UAS-Pdf, Dh31$^{\#51}$; Pdf^{01}*	-0.08589	3.5	0.0454	*
w^{1118} vs. *Dh31r$^{1/Df}$*	0.0534	2.919	0.1241	ns
w^{1118} vs. *Pdfr^{5304}*	0.2096	12.3	<0.0001	****
w^{1118} vs. *Pdfr^{5304}; Dh31r$^{1/Df}$*	0.1027	6.857	<0.0001	****
Dh31r$^{1/Df}$ vs. *Pdfr^{5304}*	0.1562	7.166	<0.0001	****
Dh31r$^{1/Df}$ vs. *Pdfr^{5304}; Dh31r$^{1/Df}$*	0.04927	2.365	0.2165	ns
Pdfr^{5304} vs. *Pdfr^{5304}; Dh31r$^{1/Df}$*	-0.1069	5.957	0.0002	***
