# Supplementary material for: Neuropeptides PDF and DH31 hierarchically regulate free-running rhythmicity in Drosophila circadian locomotor activity
Source: Sci Rep. 2019 Jan 29;9:838. doi: 10.1038/s41598-018-37107-3 (PMC6351594; doi:10.1038/s41598-018-37107-3)
Supplement: Supplementary file 3 — Figure legends [file 41598_2018_37107_MOESM3_ESM.pdf]

Supplemental data

Figure S1: The morning anticipation index and period of evening peak in each mutant: w^{1118}, *Dh31*$^{\#51}$, *Pdf*^{01}, and *Dh31*$^{\#51}$;*Pdf*^{01}.

Comparison of morning anticipation indexes (A) and time of evening peaks (B) in different genotypes. The results of a one-way ANOVA among the different genotypes are shown ($P < 0.0001$). Tukey-Kramer test results for comparisons of each genotype: ****$P < 0.0001$. The anticipation index in *Pdf*^{01} is significantly lower than that in *Dh31*$^{\#51}$;*Pdf*^{01} because *Pdf*^{01} flies exhibited a relatively higher level of locomotor activity in the middle of the night than during late night (Fig. 1D), which made the anticipation index less than 0.5 (refer to Table S1 and Materials and Methods).

Figure S2: Average daily actogram over 4 days in LD for each genotype.

Locomotor activities from days two to five in LD were averaged and plotted on the bar graph at each time point. DH31 expression in *tim-Gal4*-expressing neurons in the *Dh31* and *Pdf* double-mutant background (*tim-Gal4 > UAS-Dh31, Dh31*$^{\#51}$;*Pdf*^{01}) caused a high level of locomotor activity during the night (Fig. S2O). Because DH31 functions as a wake-promoting factor [13], it is possible that DH31 expression from *tim-Gal4*-expressing neurons promote a “wake” state in the night that resulted in the high level of nocturnal locomotor activity.

Figure S3: Double-plotted averaged actogram over 5 days in LD and 10 days in DD for each genotype.

Locomotor activities in only rhythmic flies were averaged and plotted on the double-plotted actogram, with the exception of the actogram from a single arrhythmic fly (**Q**).

Figure S4: DH31 expression in DN1ps, as well as t-DH31 or t-PDF expression in LNvs, did not rescue a severe arrhythmic phenotype in *Dh31-Pdf* double mutants.

(**A-D**) Comparison of free-running rhythms in different genotypes: w^{1118}, $Dh31^{\#51}$, Pdf^{01} and $Dh31^{\#51};Pdf^{01}$ (**A**), *UAS-Dh31/+*, *R18H11-Gal4/+* and *R18H11-Gal4 > UAS-Dh31* from the $Dh31^{\#51};Pdf^{01}$ double-mutant background (**B**), *UAS-t-Dh31/+*, *Pdf-Gal4/+*, *Pdf-Gal4 > UAS-t-Dh31* from the $Dh31^{\#51};Pdf^{01}$ double-mutant background (**C**), and *UAS-t-Pdf /+*, *Pdf-Gal4/+*, and *Pdf-Gal4 > UAS-t-Pdf* from the $Dh31^{\#51};Pdf^{01}$ double-mutant background (**D**). The data in Fig. S4A are reproduced from Fig. 1A. The proportions of rhythmic (gray bar) and arrhythmic (white bar) flies over 10 days in DD were compared via χ^2 analysis. ****P < 0.0001 and ***P < 0.001. Numbers in the bar graphs represent the number of flies.

Figure S5:

Anti-DH31 antibody immunostaining of *tim-Gal4/+; UAS-CD8::GFP/+* fly brains. Arrowhead shows DN1a. GFP (green) and DH31 (red)(A), GFP (B), and DH31 (C) are shown.

Figure S6:

Anti-VRI antibody immunostaining. *w*^{1118} (A, E), *Dh31*$^{\#51}$ (B, F), *Pdf*^{01} (C, G), and *Dh31*$^{\#51}$*;Pdf*^{01} (D, H) flies were prepared at CT13. LNvs, LNds (A-D) and DN1s (E-H) are shown.

Table S1. Morning anticipation index.

Morning anticipation index (AI) of each genotype is shown.

Table S2. Statistical analysis of morning anticipation.

Data on the statistical analysis comparing morning AIs are shown.

Table S3. Time of evening peak.

Time of evening peak in each genotype is shown.

Table S4. Statistical analysis of time of evening peak.

Data on the statistical analysis comparing time of evening peak in each genotype are shown.

Table S5. Detailed data of VRI expression.

Detailed data of the average intensity in anti-VRI immunohistochemistry are shown.

Table S6. Statistical analysis of VRI expression.

Data on the statistical analysis comparing the VRI molecular oscillations presented in Fig. 4 are shown.
